# miR-204-5p inhibits cell proliferation and induces cell apoptosis in esophageal squamous cell carcinoma by regulating Nestin

**DOI:** 10.7150/ijms.67286

**Published:** 2022-02-28

**Authors:** Honghe Luo, Weize Lv, Huayong Zhang, Chunxia Lin, Fei Li, Fangfang Zheng, Beilong Zhong

**Affiliations:** 1 Department of Thoracic Surgery, the First Affiliated Hospital, Sun Yat-sen University, Guangzhou, Guangdong 510080, China; 2 Department of Interventional Medicine, the Fifth Affiliated Hospital Sun Yat-sen University, Zhuhai, Guangdong 519000, China; 3 Department of Thyroid and Breast Surgery, the Fifth Affiliated Hospital of Sun Yat sen University, Zhuhai, Guangdong 519000, China; 4 Department of Pediatrics, the Fifth Affiliated Hospital, Sun Yat-sen University, Zhuhai, Guangdong 519000, China; 5 Department of Pharmacy, the Fifth Affiliated Hospital, Sun Yat-sen University, Zhuhai, Guangdong 519000suppl, China; 6 Department of Thoracic Surgery, the Fifth Affiliated Hospital, Sun Yat-sen University, Zhuhai, Guangdong 519000, China

**Keywords:** miR-204-5p, Nestin, esophageal squamous cell carcinoma, proliferation, apoptosis

## Abstract

Esophageal cancer (EC) is a highly malignant gastrointestinal tumor, and esophageal squamous cell carcinoma (ESCC) is one of the most common histological types of EC. MicroRNAs (miRNAs) are small noncoding RNAs closely related to tumorigenesis and tumor progression. In addition, Nestin is an intermediate filament protein (class VI) and contributes to the progression of numerous tumors. However, the correlation between miRNAs and Nestin in ESCC remains unclear. This study aimed to investigate the relationship between miR-204-5p and Nestin in ESCC. First, Nestin-related miRNAs in ESCC were explored using RNA sequencing. In ESCC tissues and cell lines, the expression of miR-204-5p was decreased detected by quantitative real-time polymerase chain reaction (qPCR), whereas Nestin protein level was upregulated identified by Western blotting (WB). Besides, Nestin was the direct target of miR-204-5p in ESCC determined via the luciferase reported assay. Moreover, miR-204-5p regulated Nestin to inhibit ESCC cell proliferation detected by the colony formation assay and promote ESCC cell apoptosis identified using the flow cytometry and TUNEL assay. Furthermore, miR-204-5p suppressed tumorigenesis *in vivo* evaluated by the murine xenograft tumor model. In conclusion, these results indicated that miR-204-5p inhibited cell proliferation and induced cell apoptosis in ESCC through targeting Nestin, which might provide novel therapeutic targets for ESCC therapy.

## Introduction

Esophageal cancer (EC) is a highly malignant gastrointestinal tumor. The most common histological types of EC are esophageal adenocarcinoma (EAC) and ESCC. In Asia, especially in China, ESCC is the most common pathological subtype 1. Even though there have been advances in diagnosis, surgical methods, radiotherapy and chemotherapy, the mortality of ESCC is still high because of the recurrence and drug resistance 2. Advances in the study of molecular biology and gene technology may help to unravel the complicated pathogenesis of EC, which includes the interaction of genetic variations and other physiological factors. So, studying the molecular mechanisms involved in the development and progression of EC is a research hotspot, and may result in the identification of vital and novel therapeutic targets.

MicroRNAs (miRNAs) are small noncoding RNAs regulating post-transcriptional gene expression 3. The RNA-induced silencing complex (RISC) is a target recognition element of mature miRNA 4. The RISC binds to its target mRNA via the 3' untranslated region (UTR), and can lead to the degradation of the mRNA, or inhibition of its translation 5, both of which result in down-regulation of protein translation. Studies have suggested that miRNAs regulate about 30% of genes, and it has been calculated that over 60% of protein-coding genes may be potential targets of miRNAs 6, 7. The genes regulated by miRNAs are involved in numerous cellular processes, and growing evidence indicates that miRNAs participate in the pathogenesis of many diseases, including cancers, nervous system diseases, autoimmune diseases, metabolic diseases, and cardiovascular diseases 8-13. Importantly, abnormal miRNA expression has been found to be closely related to tumorigenesis and tumor progression 14.

Nestin is an intermediate filament protein (class VI), and is expressed in normal and pathological cells of a number of different tissues and organs 15. Recent studies have reported that Nestin is expressed in certain malignant cells, and the high Nestin expression correlates with malignant features in some tumors. Other studies have indicated that Nestin is a novel biomarker of brain, bladder, and pancreatic cancer stem cells.^16^-^18^ To date, rare studies have revealed that Nestin positively contributes to cell proliferation and poor prognosis in esophageal squamous cancer 19. However, the function of miRNAs on the regulation of Nestin in ESCC was unclear.

Thus, the aim of our study was to identify miRNAs targeting Nestin, determine whether these miRNAs were associated with ESCC, and uncover the mechanism how miRNAs regulate Nestin in ESCC.

## Materials and Methods

### Tissue samples

Fresh ESCC tissue samples and para-carcinoma tissue samples were collected from 3 patients with ESCC treated in First Affiliated Hospital, Sun Yet-San University in 2018. The Institutional Ethics Committee of the First Affiliated Hospital of Sun Yet-San University approved this study.

### Small RNA sequencing

Trizol (Invitrogen, Carlsbad, CA, USA) was applied to extract the total RNA from the ESCC tissues according to the manufacturer's protocol. Next, the RNA molecules ranged from 18nt to 30nt were enriched by polyacrylamide gel electrophoresis (PAGE). Then both the 5` and 3' adapters were ligated to RNAs. Next, the ligation products were reverse transcripted, and the 140-160bp size products were collected to construct the cDNA library. The Agilent 2100 bioanalyzer was used to check the distribution of the size of the fragments for the library while quantitative real-time polymerase chain reaction (qPCR, TaqMan Probe) was used to quantify the library.

Subsequently, the libraries were sequenced by the BGISEQ-500 platform (BGI-Shenzhen, China). To obtain clean reads, raw reads were filtered using in-house Perl scripts. After discarding dirty reads either containing adapter or over 10% poly-N sequences and low quality reads whose Phred scores were less than 5%, all clean tags were aligned based on GeneBank database and Rfam database (11.0) to remove tRNA, scRNA, snRNA, snoRNA and rRNA. Besides, all clean tags were aligned with human reference genome (Grch37) by TopHat v2.0.9. In addition, those tags mapped to repeat sequences were also discarded. Then clean tags were blasted in miRBase database (Release 21) to identify known miRNAs.

Next, the small RNA expression level was then calculated based on transcripts per million (TPM) using the following formula: TPM=Actual miRNA counts/Total counts of clean tags×10^6^ 20. Moreover, differential expression analysis was performed using the DEGseq to determine the significance of differences of expression 21. miRNAs with a fold change ≥2 and *P* <0.05 in a comparison were determined as significant miRNAs with differently expressions.

### Bioinformatics analysis

Based on the sequences of Nestin mRNA and differently expressed miRNAs in ESCC tisssues, the candidate miRNAs targeting Nestin mRNA were predicted by *TargetScan* website and *microRNA.org* website 22, 23.

### Cell culture

ESCC cell lines including KYSE30, KYSE150, KYSE450 and the human esophageal epithelial cell (HEEC) were purchased from the Cell Bank at the Chinese Academy of Sciences (Shanghai, China). All cells were cultured with DMEM medium (Hyclone, USA) containing 100 units/mL penicillin (Hyclone, USA) and 10% fetal bovine serum (FBS) (Hyclone) at 37 °C in a 5% CO_2_ humidified incubator.

### Cell transfection and stable cell construction

In this study, pCDH-GFP+Puro lentiviral vectors were utilized for the stably transduced cell lines. All vectors used for the stably transduced cell lines were purchased from Hechuang Biotech Ltd. (Guangzhou, China). Nestin cDNA including 3'UTR was used in this study.

The KYSE30 and KYSE450 cell lines were transfected with different lentiviral vectors to establish blank expression vector-transfected stable cells (Blank vec group), miR-204-5p overexpression stable cells (miR-204-5p OE group), Nestin overexpression stable cells (Nestin OE group) and miRNA-204-5p overexpression plus Nestin overexpression stable cells (miR-204-5p OE+Nestin OE group), according to standard protocols. The stable cell lines were selected by measuring the level of green fluorescent protein (GFP) in cells. In addition, the expression of miRNA-204-5p and Nestin in the stably transfected cells was verified by qPCR and Western blotting, respectively.

### Total RNA extraction and qPCR

Total RNA from cultured cells or tissues was extracted by TRIzol reagent according to the manufacturer's instructions. For mRNA, 2 mg RNA was used for cDNA synthesis by High-Capacity RNA-to-cDNA™ Kit. For miRNA, 2 mg RNA was reverse transcribed using TaqMan miRNA assays (ABI, Forest City, CA, USA). The qPCR was performed by SYBR-based Roche Light-Cycler® 480II PCR instrument. Moreover, the relative expression levels of target genes were analyzed with 2^-ΔΔCt^ method 24. In this study, U6 and GAPDH were used as the internal references. The following primer pairs were used for qPCR: miR-204-5p forward: 5'-UUCCCUUUGUCAUCCUAUGCCU-3', reverse: 5'-CTCAACTGGTGTCGTGGA-3'; Nestin: forward, 5'-TGCGGGCTACTGAAAAGTTC-3'; reverse, 5'-GGCTGAGGGACATCTTGAG-3'; U6 forward: 5'-CTCGCTTCGGCAGCACA-3', reverse: 5'-AACGCTTCACGAATTTGCGT-3'; GAPDH forward: 5'-GGGAAACTGTGGCGTGAT-3', reverse: 5'-GAGTGGGTGTCGCTGTTGA-3'.

### Western blotting analysis

Total proteins were extracted by RIPA buffer (Cell Signaling Technology, Danvers, MA, USA). Next, proteins were separated by SDS-polyacrylamide gel electrophoresis (SDS-PAGE) and subsequently transferred onto the PVDF membrane (Millipore). Then membranes were blocked using 5% non-fat milk followed by the incubation with rabbit polyclonal anti-Nestin antibody (1:500, 19483-1-AP, Proteintech, Rosemont, IL, USA) or anti-GAPDH antibody (1:3000. IPVH00010, Millipore, Bedford, MA, USA) overnight at 4 ℃. GAPDH was used as the internal control. The next day membranes were washed by Tris-buffered saline contained 0.1% Tween20 (TBST) and incubated with anti-rabbit secondary antibody (Southern Biotech). The signals of targeted proteins were detected using chemiluminescence detection kit (Beyotime, Shanghai, China). Subsequently, densitometric analyses of bands were performed by Image J (NIH Image, Bethesda, MD, USA), and then the quantification of protein abundance was performed by normalizing the densitometric data of target protein to that of GAPDH.

### Luciferase reporter assay

The human wild-type Nestin 3'-UTR sequence or the mutated Nestin 3'-UTR sequence with the predicted target sites was amplified and subcloned into the pmirGLO Dual-Luciferase vector (HeChuang Biotech, Guangzhou, China), while miR-204-5p mimic and NC mimic were purchased from Sango (Shanghai, China). Kyse30 cells were seeded onto 24-well plates (5×10^5^ cells/well) and co-transfected with luciferase reporter vectors (0.125 μg) and miR-204-5p mimic (50 nM) or mimic negative control (50 nM) using Lipofectamine 2000 (Invitrogen).. Briefly, luciferase reporter vectors, miR-204-5p mimic or mimic negative control were added into 250 μL Opti-MEM (A solution), and 4 μL Lipofectamine 2000 was incubated with 250 μL Opti-MEM (B solution) for 5 min at room temperature (RT). Next, A solution was mixed with B solution and added into the well of 24-well plates after 20 min at RT. Then Opti-MEM was replaced with DMEM after 4 h. Finally, luciferase activity was measured by the Luciferase Assay System (Promega, Madison, WI, USA) in a bioluminescence detector (GloMax, Promega) and were normalized by the Renilla luciferase activity according to the manufacturer's protocol.

### Colony formation assay

Cell resuscitation and culture of stably transfected KYSE30 cells was performed according to the routine protocol. After trypsinization, the cells were diluted to 1×10^3^ cell/ml and inoculated on the well plate. The cells were then cultured 10 days. Next, cells were fixed by 4% paraformaldehyde solution (PFA, Invitrogen) for 1 h at RT and stained using crystal violet at RT for 30 min. Finally, the number of crystal violet-dyed colonies was calculated. The Enzyme-linked Spot Image Automatic Analyzer (AID, Germany) was used to scan and analyze the result.

### Flow cytometric analysis

Apoptosis of the stably transfected cells was evaluated by flow cytometry (BD, Bioscience, U.S.A.) using an Annexin V-FITC Apoptosis Detection Kit (KGA106, Keygen, Jiangsu, China). CellQuest software was used to analyze the results.

### TUNEL analysis

Cells were seeded onto a slide and fixed at 4℃ for 25 minutes. The slides were washed with phosphate buffer saline (PBS) twice for 5 minutes, and then treated with 70% ethanol at -20℃ overnight. Next, slides were incubated with equipment buffer for 10 min at RT. Subsequently, the slides were incubated with terminal transferase (TDT) working solution followed with 4', 6-diamidino-2-phenylindole (G3250, Promega) according to standard protocols. The slides were then observed by fluorescence microscopy (MDI6000B, Leica, Germany). The nuclei of apoptotic cells exhibited green fluorescence, and the nuclei of all cells exhibited blue fluorescence.

### Nude mice xenograft tumor model

Six-week-old male nude mice were adaptively fed for 3 days. Then 15 mice were randomly divided into 5 groups (3 mice per group). Next, mice were treated with subcutaneous injection of 2×10^6^ wild type (WT) and stable transfected KYSE 30 or KYSE450 cells 3 times as follow: 1) WT cells (NC group); 2) blank vector stable cells (Blank vec group); 3) miR-204-5p stable expression cells (miR-204-5p OE group); 4) Nestin stable expression cells (Nestin OE group); 5) miR-204-5p plus nestin stable expression cells (miR-204-5p OE+Nestin OE group).

After subcutaneous injection, tumor volume in each mouse was measured at day 4, 8, 12, 16, and 20. Then the mice were killed by cervical dislocation at day 21 after injection, and tumor size and tumor weight were measured. All animal experiments were conducted according to established guidelines for the use and care of laboratory animals, and the experiments were approved by the Beijing Deconnor Biotechnology Co.,LTD.

### Statistical analysis

All quantitative data were present as mean ± standard deviation (SD). SPSS software version 22 (IBM SPSS Inc. Chicago, IL, USA) was used to conduct the statistical analyses. Briefly, the comparation between two groups was detected using the unpaired Student's t-test. Values of *P* < 0.05 were considered to be statistically significant.

## Results

### Identification of Nestin-related miRNAs

**Figure 1:**
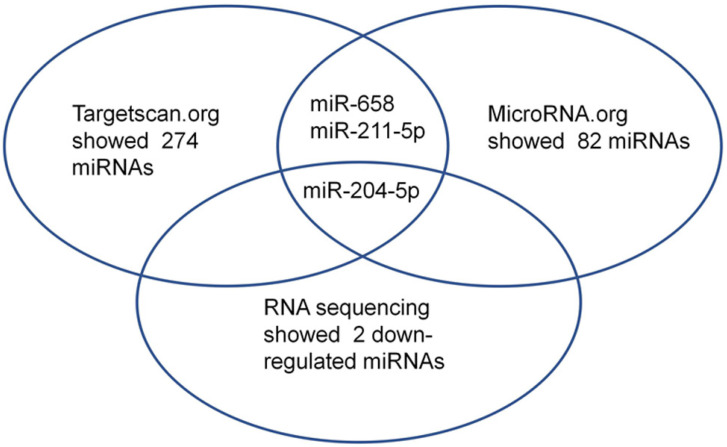
Among the microRNAs screened by *Http://www.targetscan.org* or *Http://www.mircroRNA.org* and RNA sequencing of ESCC, one candidate microRNA was overlapped.

First, small RNA libraries for paracarcinoma tissues (Normal) and ESCC tissues (Tumor) were constructed and sequenced to reveal Nestin-related miRNAs. Potential target miRNAs related to Nestin in ESCC tissues were screened with a BGISEQ500 sequencer (BGI-Shenzhen). Next, miRNAs with a fold change ≥2 and P value <0.05 in a comparison between Normal group and Tumor group were considered as significant differently expressed miRNAs. Among these differently expressed miRNAs, the candidate Nestin-related miRNAs were explored by TargetScan website and the microRNA.org website based on the sequences. The results indicated there were 19 Nestin-related miRNAs that were significantly differentially expressed between ESCC tissues and adjacent para-carcinoma tissues (Table 1). Among them, there were 2 downregulated miRNAs in ESCC tissues, including miR-204-5p and novel-miR-36 (Table 1 and Figure 1). Moreover, results screening from 2 websites (TargetScan website and the microRNA.org website) based on the free energy and score value criteria identified 3 candidate miRNAs: miR-658, miR-211-5p, and miR-204-5p (Figure 1). Taken together, miR-204-5p was the most potential target miRNA of Nestin.

In addition, previous studies have indicated that miR-204 suppresses cell proliferation, apoptosis, invasion and epithelial-mesenchymal transition (EMT) in ESCC 25-27. Based on these studies, miR-204-5p was selected for this study.

### Low miR-204-5p expression is inversely correlated with Nestin expression in ESCC tissues and cell lines

**Figure 2:**
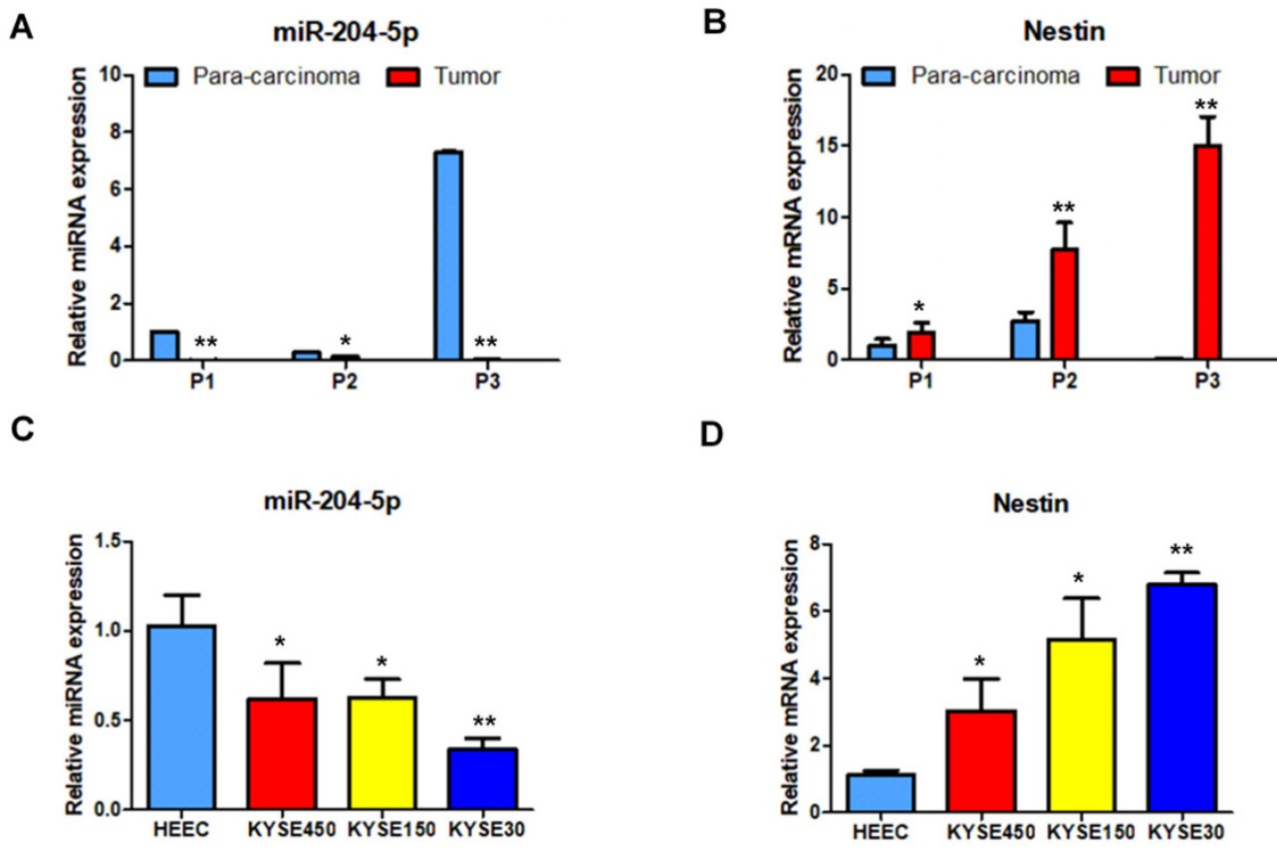
Expression of miR-204-5p and Nestin mRNA in ESCC tissues and para-carcinoma tissues, as well as ESCC cell lines and esophageal epithelial cell lines. A and B: The expression levels of miR-204-5p and Nestin mRNA in ESCC tissues and para-carcinoma tissues were determined by qPCR. C and D: The expression levels of miR-204-5p and Nestin mRNA in ESCC cells and esophageal epithelial cells were determined by qPCR. ^*^P<0.05, ^**^P<0.01 or ^***^P<0.001 compared to control group (HEEC group or para-carcinoma group). *P* values were not corrected for multiple testing.

**Figure 3:**
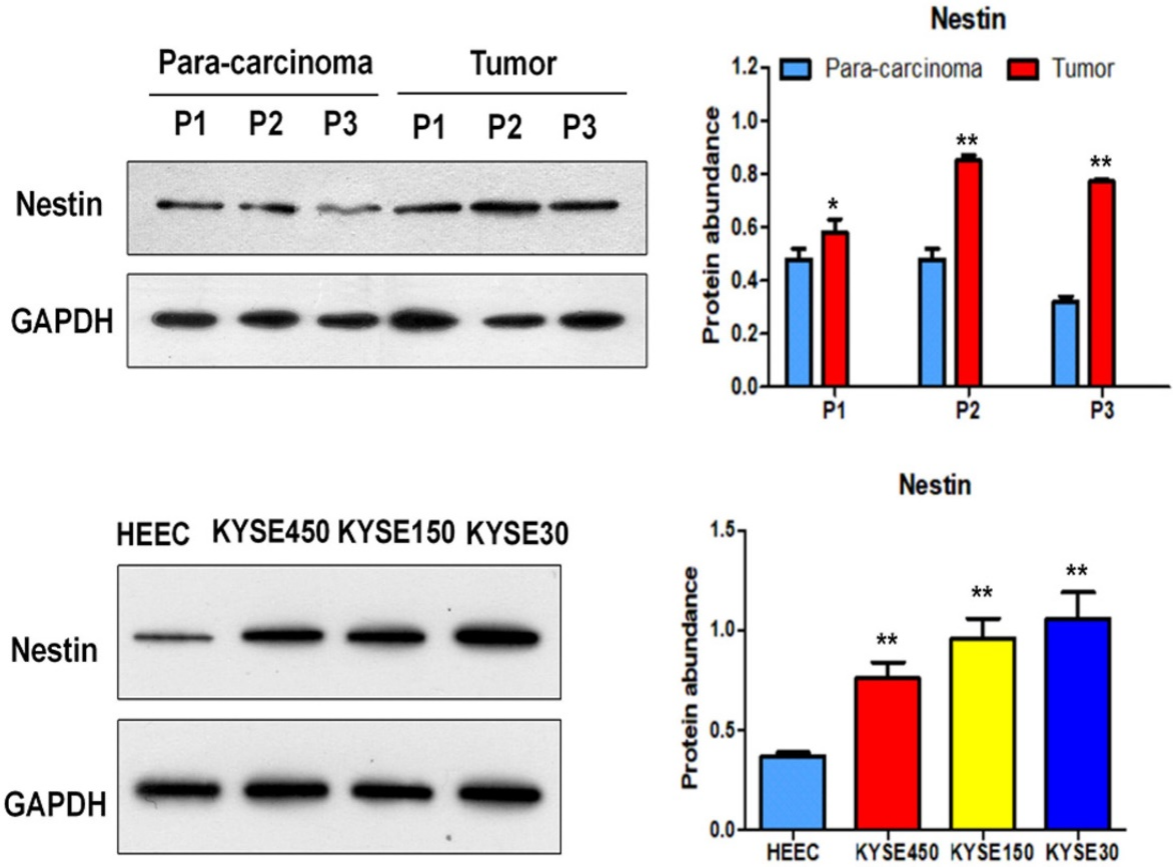
Protein expression of Nestin in ESCC tissues and para-carcinoma tissues, as well as ESCC cells lines and esophageal epithelial cell lines. A: The protein level of Nestin in ESCC tissues and para-carcinoma tissues was determined by Western blotting. B: The protein level of Nestin in ESCC cells and esophageal epithelial cells was determined by western blot. ^*^P<0.05, ^**^P<0.01 or ^***^P<0.001 compared to control group (HEEC group or para-carcinoma group). *P* values were not corrected for multiple testing.

Nestin mRNA expression exhibited a reverse trend with miR-204-5p expression in the comparison between ESCC and para-carcinoma tissues, as well as between ESCC cells and esophageal epithelial cells. In esophageal squamous cancer tissues and cell lines, Nestin mRNA expression was increased, while miR-204-5p expression was decreased; in esophageal epithelial cells and para-carcinoma tissues, Nestin mRNA expression was decreased, while miR-204-5p expression was increased, and the differences were statistically significant (Figure 2). Western blotting indicated that the level of Nestin protein in esophageal squamous cancerous cells and ESCC tissues was higher than in esophageal epithelial cells and para-carcinoma tissues (Figure 3).

### MiR-204-5p directly targets Nestin in ESCC

**Figure 4:**
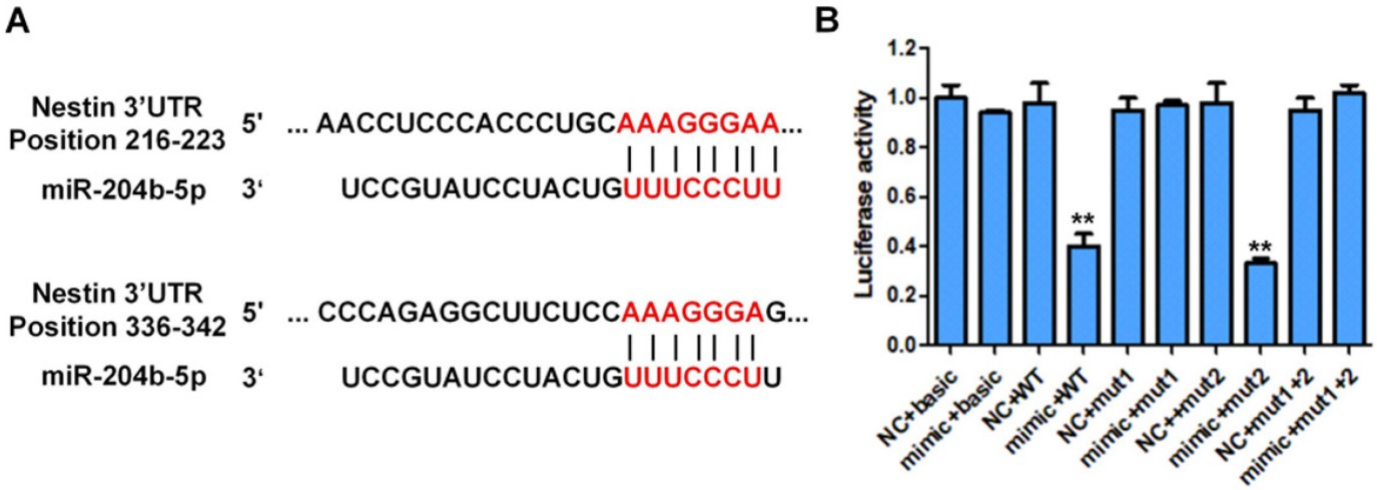
Luciferase activity of different groups of cotransfected plasmid cells with different plasmid cotransfected combination (pmirGLO, pmirGLO-Nestin WT, pmirGLO-Nestin mut1, pmirGLO-Nestin mut2, pmirGLO-Nestin mut1+2). A: Schematic representation of the nestin mRNA depicting miR-204-5p binding sites in its 3'-UTR. B: The basic group was cotransfected with pmirGLO and miR-204-5p mimic (mimic+basic group) or mimic NC ((NC+basic group). Nestin-WT (wild type) group was cotransfected with pmirGLO-nestin WT and microRNA-204-5p mimic (mimic+WT group) or NC (NC+WT group). Nestin-Mut1 group was cotransfected with pmirGLO-nestin mut1 (mutant1) with miR-204-5p mimic (mimic+mutant1 group) or NC (NC+mutant1 group). Nestin-mut2 group was cotransfected with pmirGLO-nestin mut2 and miR-204-5p mimic (mimic+mutant2 group) or NC (NC+mutant2 group). Nestin-Mut1+2 was with cotransfected with pmirGLO-nestin mut1+2 and miR-204-5p mimic (mimic+nestin-Mut1+2 group) and NC (NC+nestin-Mut1+2 group). ^*^P<0.05, ^**^P<0.01 or ^***^P<0.001 compared to NC group. *P* values were not corrected for multiple testing.

The Luciferase reporter assay was performed to investigate if miR-204-5p directly targeted Nestin in ESCC using KYSE30 cells (Figure 4). Results showed that the luciferase activity of KYSE30 cells transfected with pmirGLO-Nestin WT and miR-204-5p mimic (mimic+WT group) was dramatically decreased to that of cells transfected with blank pmirGLO and miR-204-5p mimic (mimic+basic group) (Figure 4). Besides, transfection of mimic NC had no effect on the luciferase activity of KYSE30 cells (NC+basic group and NC+WT group) (Figure 4).

Besides, the luciferase activity of KYSE30 cells transfected with pmirGLO containing the mutated binding site1 or site2 of miR-204-5p on 3' UTR of Nestin mRNA was not affected by the transfection of miR-204-5p mimic (mimic+mutant1 group, mimic+mutant2 group, mimic+ Mut1+Mut2 group) (Figure 4). These results suggested that Nestin was the target of miR-204-5p and miR-204-5p negatively regulated Nestin expression in ESCC.

### MiR-204-5p and Nestin expression in stably transfected cells and their relations

**Figure 5:**
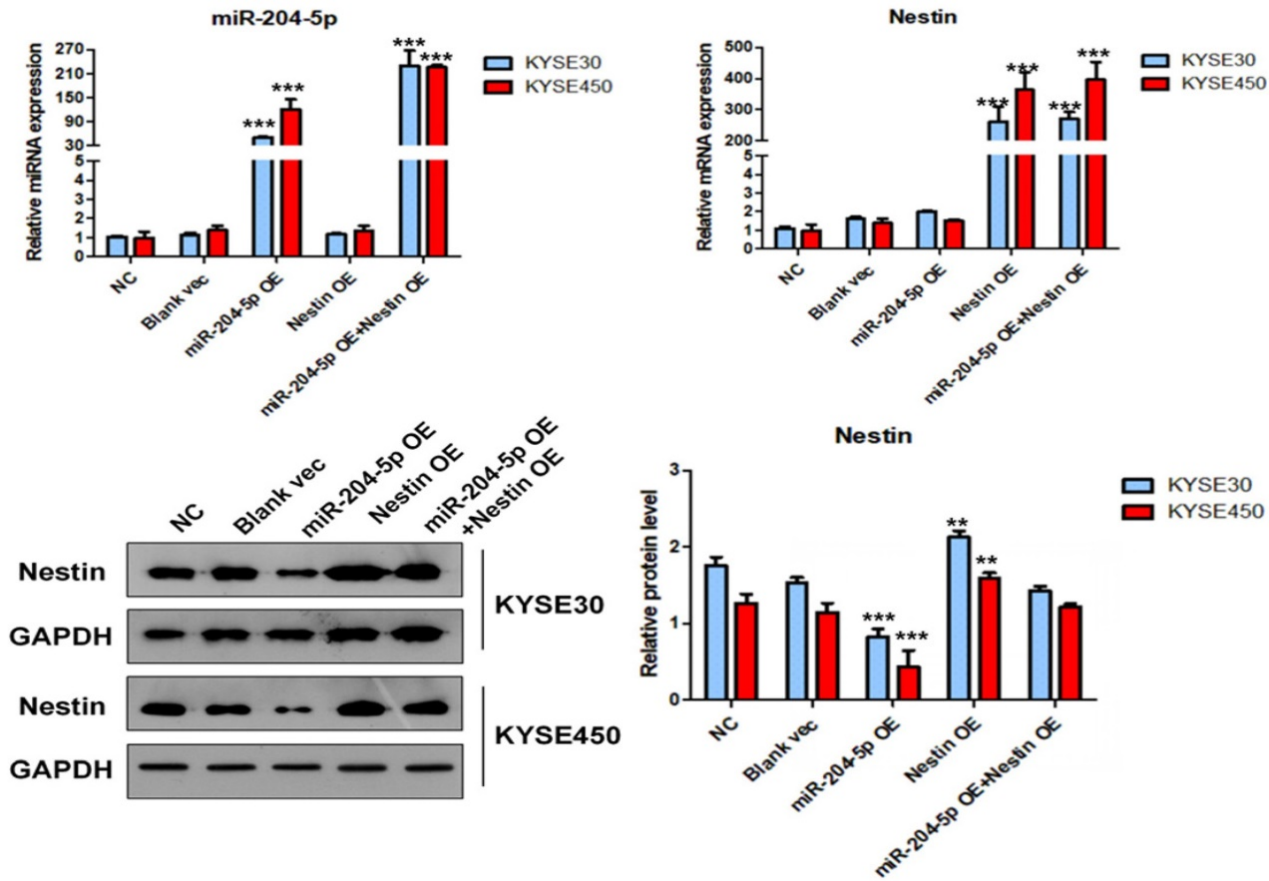
The expression level of miR-204-5p, Nestin mRNA and protein in KYSE30 and KYSE450 stably transfected cells, including NC group, Blank vec group, miR-204-5p OE group, Nestin OE group and miR-204-5p OE+Nestin OE group. A and B: The expression levels of miR-204-5p and Nestin mRNA in stably transfected cells were explored using qPCR. C: The protein levels of Nestin in stably transfected cells were determined by Western blotting, ^*^P<0.05, ^**^P<0.01 or ^***^P<0.001 compared to NC group. *P* values were not corrected for multiple testing.

Results of qPCR indicated that miR-204-5p and Nestin were stably expressed in KYSE30 and KYSE450 cells (Figure 5).

In the miR-204-5p overexpression group, the expression of miRNA-204-5p was increased, while the expression of Nestin mRNA was not affected. In the miR-204-5p overexpression +Nestin overexpression group, miR-204-5p expression was increased, but Nestin mRNA expression was not significantly reduced, indicating that miR-204-5p could not inhibit Nestin mRNA expression. Usually, miRNA inters with target mRNA to suppress the translation of mRNA but not degrade mRNA 28. Thus, Western blotting was utilized to identify the effect of miR-204-5p on Nestin protein.

Western blotting results indicated that the level of Nestin protein was significantly reduced in the miR-204-5p OE group but was relatively higher in the miR-204-5p overexpression +Nestin overexpression group. This result suggests that miR-204-5p inhibits Nestin protein expression in KYSE30 and KYSE450 cells, and the Nestin overexpression reverses miR-204-5p inhibition.

### MiR-204-5p inhibits ESCC cell proliferation by targeting Nestin *in vitro*

**Figure 6:**
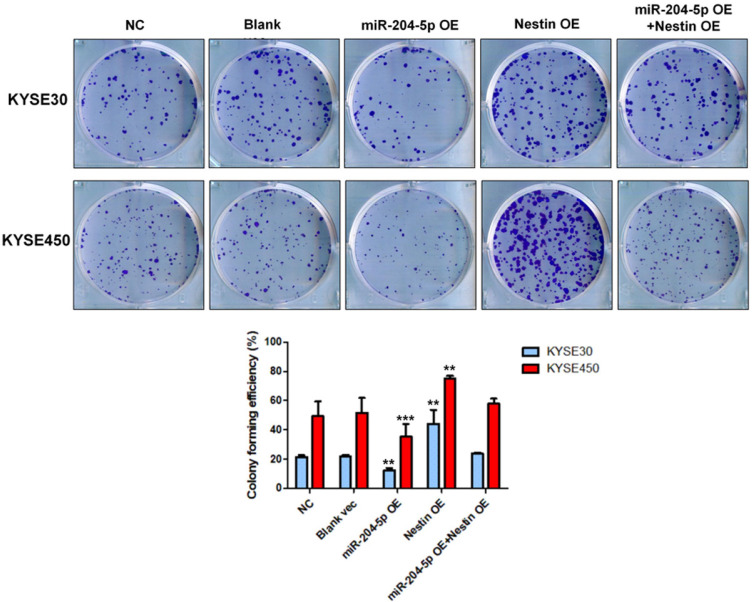
Colony formation assay was performed in KYSE30 and KYSE450 stably transfected cells, including NC group, Blank vec group, miR-204-5p OE group, Nestin OE group and miR-204-5p OE +Nestin OE group. ^*^P<0.05, ^**^P<0.01 or ^***^P<0.001 compared to NC group. *P* values were not corrected for multiple testing.

Results of colony formation assay indicated that the number of cell clones was significantly increased in Nestin overexpression group, while that was dramatically reduced in the miR-204-5p overexpression group compared with negative control group, vector control cells, and the miR-204-5p overexpression +Nestin overexpression group (Figure 6). These results suggested that miR-204-5p inhibited the clone formation ability of KYSE30 and KYSE450 cells, while Nestin overexpression reversed miR-204-5p inhibition.

### MiR-204-5p induces cell apoptosis of ESCC by targeting Nestin *in vitro*

**Figure 7:**
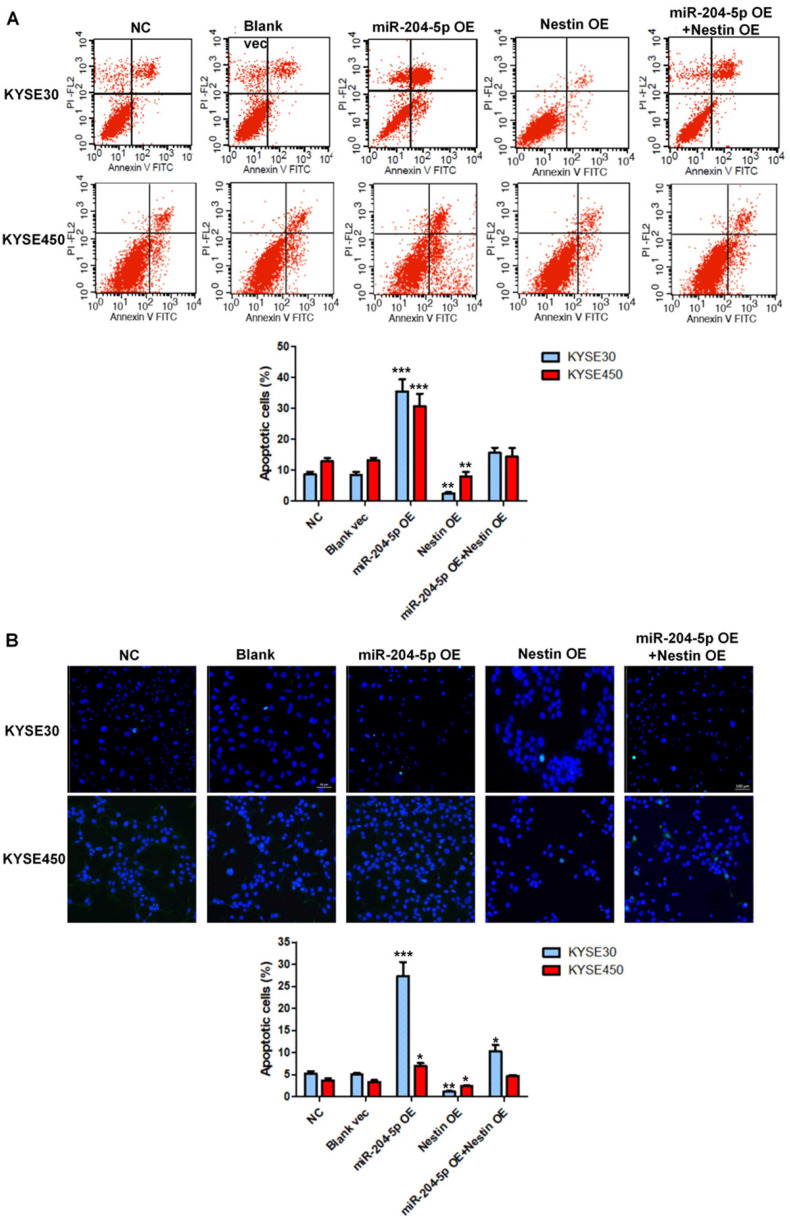
Cell apoptosis in the KYSE30 and KYSE450 stably transfected cells were analyzed by flow cytometry (A) and TUNEL assay (B).^ *^P<0.05, ^**^P<0.01 or ^***^P<0.001 compared to NC group. *P* values were not corrected for multiple testing.

Cell apoptosis in stably transfected KYSE30 cells and KYSE450 cells was analyzed by flow cytometry. Results showed that miR-204-5p overexpression significantly induced apoptosis of KYSE30 cells and KYSE450 cells whereas Nestin overexpression suppressed cell death of KYSE30 cells and KYSE450 cells (Figure 7A). Consistent with the results of flow cytometry, the number of TUNEL-positive cells was highest in the miR-204-5p overexpression group while that was lowest in Nestin overexpression group (Figure 7B). Moreover, Nestin overexpression could abolish the effect of miR-204-5p overexpression on cell apoptosis (Figure 7A and B). These results suggested that miR-204-5p could induce apoptosis in KYSE30 and KYSE450 cells through regulating Nestin.

### MiR-204-5p inhibits xenograft tumor growth* in vivo*

**Figure 8:**
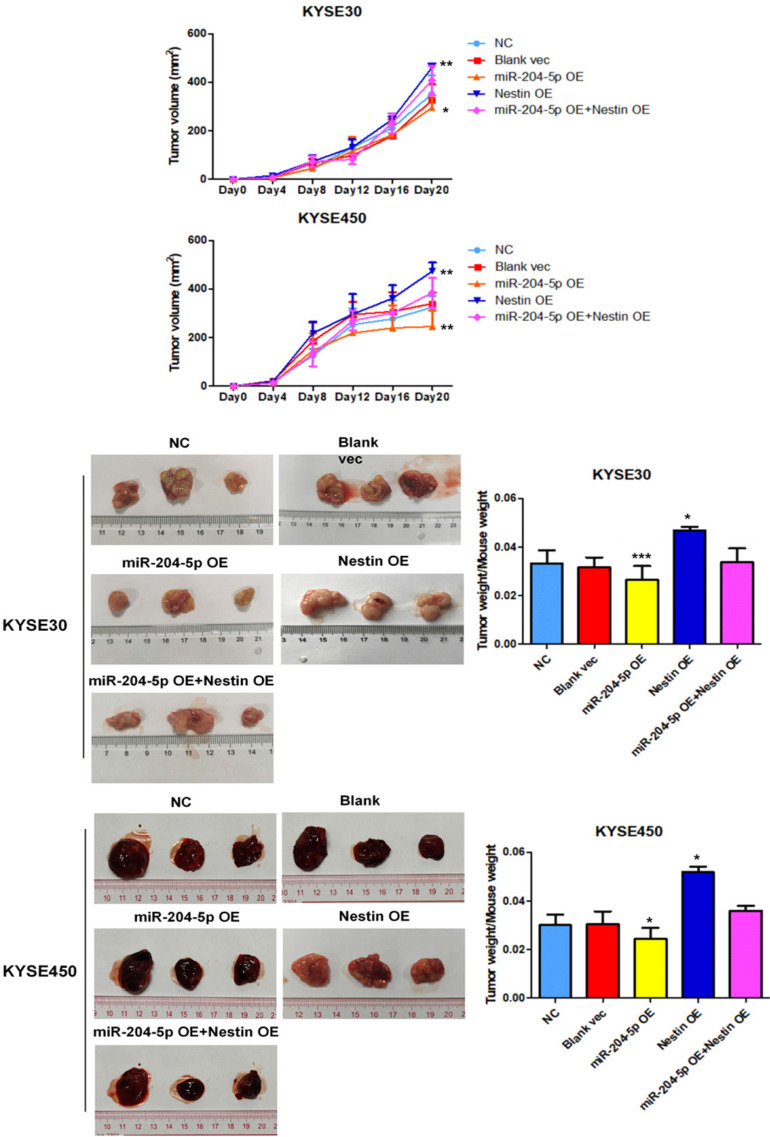
The effect of miR-204-5p on tumor growth was assessed by a nude mice xenograft tumor model. A: Growth curve of tumor volumes were calculated. Data were shown as mean ± SD. B: Photographs of tumors size and weight obtained from the different groups of nude mice. ^*^P<0.05, ^**^P<0.01 or ^***^P<0.001 compared to NC group. *P* values were not corrected for multiple testing.

A murine xenograft tumor model was used to assess the effect of miR-204-5p on tumor growth. Tumor volume was measured at set time points after injection with stably transfected KYSE30 and KYSE450 cells. Of the 5 groups, tumor volume was the smallest in the miR-204-5p overexpression group while that was largest in the Nestin overexpression group (Figure 8A). The mice were killed on day 21 after injection, and then tumor size and weight were measured. Of the 5 groups, the weight of the group injected with miR-204-5p overexpression stable cells was the lightest while that was heaviest in the group injected with Nestin overexpression stable cells (Figure 8B). Furthermore, Nestin overexpression reversed the effect of miR-204-5p overexpression on suppressing the tumor growth (Figure 8A and B). These results suggest that xenograft tumor growth is inhibited by miR-204-5p *in vivo*, and the inhibition is attenuated by Nestin overexpression.

## Discussion

ESCC is a common and prevalent malignant tumor of digestive system worldwide, especially in China. The disease is associated with a poor prognosis and high mortality, and thus researches are focused on new and novel treatments. Targeted therapies have been shown to be promising for many cancers, such as lung cancer and breast cancer and so on. It is implied that studies of the pathogenic mechanisms of esophageal cancer may lead to the development of novel targeted treatments.

Nestin is originally found to be expressed in progenitor cells of the nervous system, and subsequently its expression has been found to be elevated in several different malignant tumors. Our prior study showed that Nestin is expressed in ESCC, and 32 of 93 cases of ESCC patients (34.4%) are positive for Nestin expression 19. This finding is similar with that of Shinichiro et al who reported that Nestin is expressed in 35.5% of lung cancers. At the same time, Nestin is also detected in Eca-109 and TE-1 cells.^29^

Our prior study has confirmed the association between Nestin and ESCC, and the results of that study lead us to 3 conclusions: 1) Nestin can be expressed in ESCC tissues and cell lines; 2) Nestin-positive ESCC is associated with a poor prognosis; 3) A positive correlation between Nestin phenotype and tumor cell proliferation indexes (Ki67 and PCNA) indicated that Nestin may be involved in the malignant proliferation of ESCC. However, only one study has revealed that Nestin positively contributes to cell proliferation and poor prognosis in ESCC 19. Thus, the current study was performed to explore the mechanism how Nestin regulates the proliferation of ESCC cells.

In recent years, several studies have revealed the associations between miRNAs and esophageal carcinoma. For example, it has been reported that miR-186 regulates cell proliferation and apoptosis in human ESCC by targeting SKP2 30. MiR-134 suppresses the development of ESCC through blocking MAPK pathway via targeting PLXNA1.^31^ Moreover, circular RNA ciRS-7 enhances the growth and metastasis of ESCC through inhibiting the effect of miR-7 on HOXB13 32. To identify Nestin-related miRNAs in ESCC, differently expressed miRNAs in ESCC tissues compared with paracarcinoma tissues were identified by small RNA sequencing. Paracarcinoma tissues are normal tissues and serve as controls for tumor tissues to reveal differently expressed genes in tumor tissues. Subsequently, Nestin-related miRNAs identified by small RNA sequencing were compared with results screening from 2 websites (TargetScan website and the microRNA.org website) to further demonstrate the potential Nestin-related miRNAs. Results found that the number of Nestin-related miRNAs predicted by the two online resources was different (TargetScan website showed 274 miRNAs while microRNA.org website showed 82 miRNAs), which might be due to the different rules utilized by different online resources to predict the interaction between target mRNA and miRNA. Thus, these data suggested that the prediction of miRNA-mRNA interaction should be based on overlapped information obtained from different resources.

To date, the role of miR-204-5p in the development of esophageal cancer is still not clear. More and more researches reveal that miR-204-5p plays an important role in many diseases, especially in malignant tumors. Wang *et al.* investigated the expression and functional roles of miR-204-5p in OSCC 33. They found that miR-204-5p could enhance OSCC cell proliferation and metastasis by targeting CXCR4. In addition, some studies have shown that miR-204-5p is regulated by long noncoding RNA (lncRNA). Yu *et al*. reported that LncRNA TUG1 positively regulated the expression via sponging miR-204-5p to promote osteogenic differentiation in calcific aortic valve disease (CAVD) 34. Recently, the study of miR-204-5p in ESCC has also been reported. Tang *et al.* investigated the potential role of miR-204-5p in ESCC. They found that miR-204-5p functioned by directly targeting IL-11 26. However, the molecular mechanism and signal transduction mechanism of miR-204-5p in ESCC are still unknown. In the current study, we demonstrated for the first time that miR-204-5p regulates ESCC via targeting Nestin.

Our results suggest that miR-204-5p targets the Nestin 3'UTR, and the target recognition element of RISC bound to the 3'UTR of targeted mRNA. Because miRNA-204-5p is not tightly bound to Nestin, it only inhibits Nestin protein translation, but does not degrade Nestin mRNA, resulting in down-regulation of protein expression. As an upstream regulator of Nestin, miR-204-5p may inhibit cancer cell proliferation and induce cancer cell apoptosis in esophageal squamous cancer cells.

## Conclusions

The results of this study showed that miR-204-5p may target Nestin mRNA, and miR-204-5p could inhibit the proliferation and induced apoptosis of ESCC cells by regulating Nestin. These results suggested that miR-204-5p maybe a key regulator of Nestin in ESCC and targeting miR-204-5p might be a novel treatment for ESCC.

## Figures and Tables

**Table 1:** Results of related Nestin's target microRNAs from genetic detection in ESCC tissues.

miRNA	Symbol	Read count (NORMAL)	Read count (TUMOR)	log_2_ Ratio (TUMOR/NORMAL)	Up down regulation
hsa-miR-204-5p	NES	54471	3712	-3.689	DOWN
has-novel-miR-36	NES	82	0	-7.172	DOWN
hsa-miR-4443	NES	43	76	1.008	UP
hsa-miR-17-3p	NES	531	953	1.030	UP
hsa-miR-432-5p	NES	421	848	1.197	UP
hsa-miR-1306-5p	NES	72	154	1.283	UP
hsa-miR-1294	NES	52	121	1.404	UP
hsa-miR-1268b	NES	41	99	1.458	UP
hsa-miR-4446-3p	NES	29	84	1.720	UP
hsa-miR-4516	NES	111	328	1.750	UP
hsa-miR-1293	NES	5	21	2.256	UP
hsa-miR-6720-5p	NES	11	61	2.657	UP
hsa-miR-940	NES	60	357	2.759	UP
hsa-miR-1268a	NES	386	2309	2.767	UP
hsa-miR-345-5p	NES	8385	62544	3.085	UP
hsa-miR-544b	NES	0	10	4.508	UP
hsa-miR-509-5p	NES	1	26	4.886	UP
hsa-miR-1273h-5p	NES	0	14	4.993	UP
hsa-miR-3194-5p	NES	0	19	5.434	UP

The read count and log_2_ Ratio of normal and tumor was calculated for the difference between paracancerous and cancerous tissues of ESCC through RNA sequencing. Abbreviation: MiRNA: microRNA; NES: Nestin.

## Abbreviations

EC: esophageal cancer
EAC: esophageal adenocarcinoma
ESCC: esophageal squamous cell carcinoma
RISC: RNA-induced silencing complex
UTR: untranslated region
HEEC: human esophageal epithelial cell lines
qPCR: quantitative real-time polymerase chain reaction
RT-PCR: reverse transcription polymerase chain reaction
GFP: Green fluorescent protein
SDS-PAGE: sodium dodecyl sulfate polyacrylamide gel electrophoresis
PVDF: polyvinylidene fluoride
GAPDH: glyceraldehydes-3-phosphate dehydrogenase
TUNEL: terminal deoxynucleotidyl transferase mediated nick end labeling
PBS: phosphate buffer saline
NC: negative control
PCNA: proliferating cell nuclear antigen
CDK5: cyclin dependent kinase-5
